# Geospatial analysis of neonatal mortality in north-eastern India: a multilevel Bayesian approach

**DOI:** 10.4178/epih.e2025021

**Published:** 2025-04-27

**Authors:** Vidhi Jain, Kh. Jitenkumar Singh, Deboshree Das, Shefali Gupta, Gunjan Singh

**Affiliations:** 1 Amity School of Applied Sciences, Amity University Lucknow Campus, Lucknow, India; 2 Indian Council of Medical Research, New Delhi, India; 3 Indian Council of Medical Research - National Institute for Research in Digital Health and Data Science, New Delhi, India; 4 Department of Statistics, Amity School of Applied Sciences, Amity University Lucknow Campus, Lucknow, India

**Keywords:** Epidemiology, Public health, Logistic regression, Neonatal mortality, Geographically weighted regression, Spatial analysis

## Abstract

**OBJECTIVES:**

Neonatal mortality remains a significant public health issue in India. This study investigates spatial patterns and contributing factors to neonatal mortality in the north-eastern states, identifying hotspot regions and spatial variations.

**METHODS:**

A sample of 34,222 mothers from India’s National Family Health Survey (NFHS-5, 2019-21) in the north-eastern states was analysed. Descriptive and bivariate analyses were conducted alongside Bayesian multilevel logistic regression using integrated nested Laplace approximation to model neonatal mortality. Spatial hotspot analysis using Getis-Ord Gi* statistics identified clusters of high neonatal mortality, while geographically weighted regression (GWR) was used to examine spatial variations in the relationships between neonatal mortality and contributing factors.

**RESULTS:**

The neonatal mortality rate in the north-eastern states declined from 45 to 21 per 1,000 live births (NFHS-1 to NFHS-5) but remains higher than the national average. Assam reported the highest mortality (42.16%), whereas Sikkim had the lowest (0.87%). Higher mortality was observed among male infants, mothers with advanced age, low maternal education, and mothers who attended less than 5 antenatal care (ANC) visits. Spatial analysis identified hotspots in Assam, Meghalaya, and Tripura. GWR indicated that areas with less than 5 ANC visits had the strongest association with neonatal mortality. Bayesian multilevel analysis highlighted spatial variations of up to 51% across districts in northeast India.

**CONCLUSIONS:**

This study underscores spatial disparities in neonatal mortality across north-eastern India. Addressing childcare practices and healthcare access in hotspot regions is essential for improving new-born health outcomes. The findings provide critical insights for policymakers to develop targeted interventions aimed at reducing neonatal mortality in these underserved areas.

## GRAPHICAL ABSTRACT

[Fig f6-epih-47-e2025021]

## Key Message

Neonatal mortality in Northeastern India is disproportionately high in rural areas, especially among male infants, those born to mothers with advanced age or low educational levels, and households with limited access to antenatal care or wealth. This study utilizes advanced spatial modelling techniques to identify regional disparities and hotspot clusters of neonatal mortality. Findings highlight the need for targeted public health interventions in specific districts, emphasizing the importance of improving maternal health services and economic conditions. The study contributes valuable insights into the epidemiology of neonatal mortality, helping guide policy decisions aimed at reducing health inequities in these underserved regions.

## INTRODUCTION

Neonatal mortality management is a critical aspect of public health, reflecting the overall effectiveness of maternal and child healthcare systems. The first 28 days of life constitute the most vulnerable period for neonatal survival. Many neonatal deaths can be prevented through improved maternal care, skilled birth attendance, and early neonatal interventions. Addressing neonatal mortality also aligns with global health goals, such as the Sustainable Development Goals, which aim to reduce preventable child mortality and improve healthcare equity, particularly in high-risk regions. Globally, around 2.8 million of the total under-5 mortalities were neonatal deaths in 2013 [1-3]. The World Health Organization defines neonatal mortality as “mortality occurring within the first 29 days of life among live births” [4]. Neonatal mortality is further divided into early neonatal mortality (within 0 to 7 days after birth) and late neonatal mortality (between 7 to 28 days after birth) [1].

Despite a global decline in neonatal mortality rates (NMRs) from 36.6 per 1,000 live births in 1990 to 18.4 per 1,000 live births in 2013 [1,2], the primary causes of neonatal mortality—such as preterm birth complications, neonatal encephalopathy from birth asphyxia, neonatal sepsis, and other infections—remain major concerns [1,2]. Global estimates indicate significant regional variations in neonatal mortality causes. Only 7% of neonatal deaths in high-income countries result from infectious diseases, while around 27% of total neonatal deaths occur in sub-Saharan Africa, and 23% occur in Southern Asia [1].

Neonatal mortality remains a major public health challenge, especially in low-middle to middle-income countries such as India [5]. In 2021, the global NMR was 17 per 1,000 live births, whereas India’s National Family Health Survey-5 (NFHS-5) reported a higher national NMR of 25 per 1,000 live births [6]. However, this figure masks significant state-level differences. North-eastern states exhibit even higher rates than more developed states like Kerala and Tamil Nadu, primarily due to disparities in healthcare access and lower antenatal care (ANC) coverage. Socioeconomic factors, including maternal education, household wealth, and access to safe drinking water and sanitation, further widen these disparities. Additionally, cultural and geographical barriers in tribal and remote areas of the northeast restrict access to essential maternal and child health services. Understanding these regional variations is crucial for targeted policy interventions aimed at reducing neonatal deaths and improving overall child survival rates in India. Nonetheless, over the past 25 years, India has made significant progress, halving neonatal mortality among infants under 1 month of age [7]. The home-based newborn care [8] initiative aims to decrease neonatal mortality and morbidity rates, particularly in rural and remote areas where healthcare services are limited. Most primary health centres and community health centres in India are now equipped with new-born care corners in labour rooms and obstetric operation theatres, providing essential new-born care and services [9]. A nationwide network of facility-based new-born care has been established, comprising 14,135 new-born care corners, 1,810 new-born stabilisation units, and 548 special new-born care units catering to sick and small new-borns [10]. Furthermore, initiatives such as *Janani Shishu Suraksha Karyakram, Janani Suraksha Yojana*, and *Pradhan Mantri Surakshit Matritva Abhiyaan* under the National Health Mission ensure that all pregnant women and infants receive free delivery, medications, diagnostics, treatment, nutrition, and transportation to and from healthcare facilities [10].

According to the NFHS-5 survey, neonatal mortality varies across Indian states due to wide variations in healthcare service accessibility and availability [11]. Some north-eastern states exhibit higher NMRs than the national average. The geographical features of north-eastern states also impact healthcare access; mountainous terrains often pose challenges to healthcare connectivity [12]. This study utilized data from NFHS-5 to examine factors associated with spatial variations in neonatal mortality across the north-eastern states.

The NMR serves as a key indicator of new-born health. The north-eastern states, among India’s most remote and underdeveloped regions, face significant hurdles. Furthermore, only a limited number of studies—particularly in northeast India—have highlighted how neonatal mortality and its determinants vary spatially. Thus, identifying hotspots and assessing the magnitude of clustering in neonatal mortality within these states is critical. Additionally, understanding spatial variations in the relationships between neonatal mortality and its contributing factors can help policymakers formulate targeted interventions. The aims of this study are: (1) to observe levels and trends in NMRs, (2) to identify spatial hotspot clustering, and (3) to assess factors associated with varying space.

## MATERIALS AND METHODS

### Source of data

The data for this study were sourced from the NFHS-5 (2019-21), a comprehensive survey conducted across representative households in India. NFHS provides critical insights into demographics, health indicators, and nutritional status, managed by the Ministry of Health and Family Welfare (MOHFW) and coordinated by the International Institute for Population Sciences (IIPS). NFHS-5 used a stratified multi-stage sampling design, selecting primary sampling units (PSUs) in rural areas and census enumeration blocks (CEBs) in urban areas. In each PSU or CEB, 22 households were randomly chosen. A sample of 34,222 mothers from NFHS-5 (2019-21) in the north-eastern states was analysed. The analysis focused on live-born neonates (0-28 days), excluding stillbirths, miscarriages, and cases with missing or inconsistent data on key variables. Women responded to survey questions about all children born to them in the past 5 years. This study employed spatial modelling to analyse neonatal mortality patterns in the north-eastern states of India.

### Outcome measurements

The primary outcome measure in this study was neonatal mortality in the north-eastern states of India. Neonatal mortality serves as a critical indicator of population health and healthcare accessibility, particularly in evaluating maternal and child health initiatives [13]. Neonatal mortality refers to the death of new-borns within the first 28 days of life—a critical period determining infant survival and long-term health. It is typically measured as the NMR, defined as the number of neonatal deaths per 1,000 live births in a given year or population [4].

This study used NFHS-5 (2019-21) data to estimate regional NMRs by applying weighted estimates, ensuring representativeness at both the state and district levels.


$$NMR=\frac{Number\;of\;neonatal\;deaths}{Total\;live\;births}x\;1,000$$

Spatial analytical techniques were applied to identify regional variations and hotspots of neonatal mortality across the north-eastern states of India.

### Predictor variables

**Figure 1.**
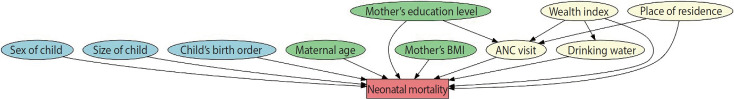
Causal relationships between predictors and neonatal mortality. BMI, body mass index; ANC, antenatal care.

Predictor variables included in this study were selected after reviewing existing literature based on their established influence on neonatal mortality [14]. Key variables included the sex of the child, size of the child, child’s birth order, maternal age, mother’s educational level, mother’s body mass index (BMI), ANC visits, drinking water quality, wealth index, and place of residence. To model causal pathways among these predictors with neonatal mortality as the outcome, a directed acyclic graph was generated (Figure 1).

### Statistical analysis

#### Trends in neonatal mortality in the north-eastern states

Trends in neonatal mortality in India and the north-eastern states were estimated using data from NFHS-1 (1992-93) to NFHS-5 (2019-21). Univariate analysis was performed using R software to estimate neonatal mortality prevalence for each NFHS round.

#### Bivariate analysis and Bayesian multilevel logistic regression

Descriptive statistics and chi-square tests of association were used to present sample characteristics and study the associations between selected socio-demographic, maternal, and child characteristics and neonatal mortality across the north-eastern states. Bayesian multilevel logistic regression was utilised to estimate adjusted odds ratios of neonatal mortality. Multilevel modelling is particularly suitable when examining relationships between variables across different hierarchical levels. This method is appropriate given that NFHS data have a hierarchical structure, where neonatal death (outcome) is measured at the individual level (lowest level); individual women are nested within households, households within communities, communities within districts, and districts within states. Multilevel analyses account for variables at different levels of analysis, thus enabling assessment of macro-level variables alongside the typically used individual-level factors [15].

The Bayesian approach for estimating unknown model parameters provides accurate predictive measures using less data points, with the added ability to quantify uncertainty around parameter estimates. Additionally, it predicts full posterior distributions for each parameter, integrating prior information with observed data. In contrast, traditional regression estimates parameters through maximum likelihood estimation, which does not fully account for uncertainty beyond standard errors. The model estimated the intraclass correlation coefficient (ICC), measuring the degree of clustering within groups or classes. A high ICC value indicates clustering of neonatal deaths at higher hierarchical levels.

#### Hotspot analysis using the Getis-Ord Gi* statistic and heat map

Hotspot analysis identifies statistically significant hotspots and coldspots by aggregating occurrence points into polygons or convergence points based on calculated distances. The analysis groups features where similar high (hot) or low (cold) values form clusters. The hotspot analysis tool calculates the Getis-Ord Gi* statistic for each feature in the dataset. The resultant Z-score indicates areas where features with either high or low values spatially cluster. Statistically significant hotspots occur where features with high values are surrounded by other high-valued features, resulting in a high Z-score and a low p-value. Conversely, a low negative Z-score with a small p-value indicates a significant coldspot. A Z-score close to 0 suggests no spatial clustering [16]. Additionally, heatmaps were generated to visualise the relative intensity of neonatal mortality within identified hotspots across the north-eastern states. A heatmap is a 2-dimensional visualisation tool using colour gradients to represent the magnitude of dataset values.

#### Geographically weighted regression

The study applied geographically weighted regression (GWR) when explanatory variables demonstrated both strong and weak predictor relationships among clusters. GWR is an extension of ordinary least squares regression, examining spatial variability in relationships between neonatal mortality and explanatory variables. GWR generates a local regression equation for each cluster using data from nearby clusters. Consequently, each spatial unit has its own specific coefficient value, making the model results more reflective of local situations and preserving local relationship characteristics between variables [17]. Maps displaying the regression coefficients (β coefficients) associated with each independent variable can inform targeted interventions. Additionally, multicollinearity was assessed using the variance inflation factor (VIF). The VIF diagnostic test evaluated multicollinearity among explanatory variables [18]. No significant multicollinearity was detected (VIF<5). Spatial analysis and visualisation employed software including Anaconda3 (Python distribution), R version 4.3.2 (R Foundation for Statistical Computing, Vienna, Austria), GeoDa (GeoDa Center for Geospatial Analysis and Computation, Arizona State University, Tempe, AZ, USA), MGWR 2.2 (Spatial Analysis Research Group, Arizona State University, Tempe, AZ, USA), and Stata version 18 (StataCorp., College Station, TX, USA).

### Ethics statement

The study used secondary data extracted from the most recent NFHS-5 survey. Because the dataset is openly available in the public domain for research purposes, ethical approval was not required. The data are accessible via the Demographic and Health Surveys website [6].

## RESULTS

### Trends in the neonatal mortality rate in north-eastern states and India

**Figure 2.**
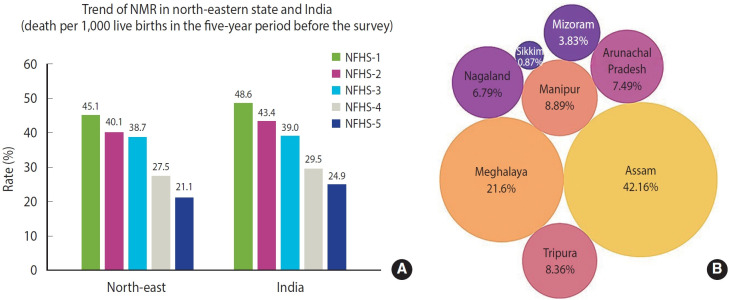
(A) Trends in the neonatal mortality rate (NMR) from NFHS-1 to NFHS-5 in the north-eastern states and in India. (B) Circular pack diagram for neonatal mortality. NFHS, National Family Health Survey.

The trends depicted in Figure 2A illustrate NMR in the north-eastern states compared with India as a whole. In the north-eastern states, neonatal mortality decreased from 45.1 deaths per 1,000 live births (NFHS-1) to 21.1 deaths per 1,000 live births (NFHS-5). During the same period, India’s overall neonatal mortality decreased from 48.6 deaths per 1,000 live births (NFHS-1) to 24.9 deaths per 1,000 live births (NFHS-5). Figure 2B presents a circular pack diagram where each circle’s size corresponds proportionally to the percentage of mortality: larger circles represent higher percentages of neonatal mortality, and smaller circles indicate lower mortality percentages. Analysis showed a total of 574 neonatal deaths in the north-eastern states, with Assam reporting the highest at 242 deaths (42.16%) and Sikkim the lowest at 5 deaths (0.87%).

**Figure 3.**
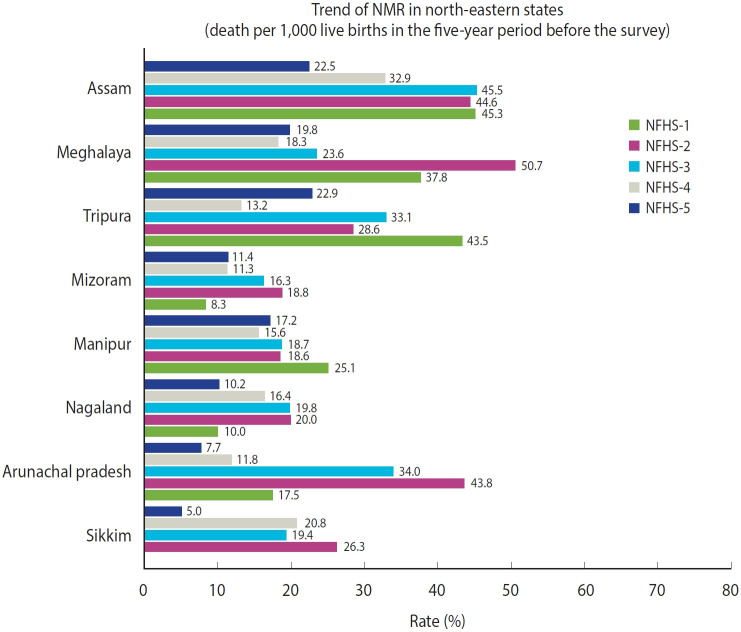
Trend of neonatal mortality rate (NMR) from NFHS-1 to NFHS-5 in north-eastern states, India. NFHS, National Family Health Survey.

Figure 3 presents state-specific trends, clearly indicating that Assam recorded the highest neonatal mortality in NFHS-1, NFHS-3, and NFHS-4; Meghalaya in NFHS-2; and Tripura in NFHS-5.

### Bivariate analysis of neonatal mortality in the north-eastern states and predictor variable

The bivariate analysis indicated that neonatal mortality was more common among male infants compared to females and infants of below-average size at birth compared to average-sized infants. Additionally, lower mortality was noted among third-order births compared to first-order births. Neonatal mortality was notably higher among mothers aged 30-49 years at childbirth and mothers with only primary education. Furthermore, mothers with low BMI and less than 5 ANC visits had higher NMRs compared to their counterparts. Households classified in the lowest wealth quintile and those with inadequate drinking water facilities also experienced elevated neonatal mortality. Moreover, neonatal mortality was higher in rural areas than in urban areas.

As shown in Table 1, the odds of neonatal mortality were notably higher among mothers aged 30-49 years at childbirth (odds ratio, 10.68). Multicollinearity among predictor variables was not observed; the highest VIF recorded in the study was 1.02, indicating very low multicollinearity among the selected predictor variables.

### Heatmap and spatial hotspot clustering of neonatal mortality

**Figure 4.**
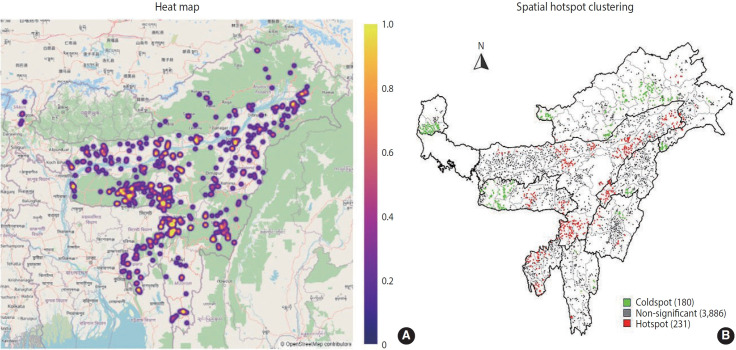
(A) Heat map of neonatal mortality. (B) Spatial hotspot clustering of neonatal mortality.

Figure 4A illustrates a heatmap of neonatal mortality based on NFHS-5 data. The colour gradient ranges from blue for the lowest mortality rates to yellow for the highest, with mid-range values depicted by shades of light red and yellow [19]. The results based on regional population density indicate that NMRs were highest in Meghalaya among the north-eastern states.

Figure 4B provides a detailed geographic distribution of neonatal mortality hotspots in the region. Spatial analysis identified the highest concentration of neonatal mortality clusters in Assam, followed by Meghalaya, Nagaland, Arunachal Pradesh, Manipur, Tripura, Mizoram, and Sikkim. Notably, hotspots appeared across various districts, with Hailakandi in Assam being the most significant. Other districts exhibiting hotspot clusters included West Khasi Hills in Meghalaya, Peren in Nagaland, Namsai in Arunachal Pradesh, Chandel in Manipur, and North Tripura district in Tripura. Additional hotspot clusters were observed in Lawngtlai district of Mizoram and the North District of Sikkim.

### Geographically weighted regression results

The results of the GWR analysis indicated that attending less than 5 ANC visits exhibited the strongest spatial relationship with NMRs, evidenced by the lowest Akaike information criterion value (12,177.701) and highest adjusted R² (0.005). This suggests that areas with higher concentrations of mothers who attended less than 5 ANC visits tended to experience higher neonatal mortality, although the overall explanatory power of the model remained relatively low. Other factors, including lower levels of primary education and advanced maternal age, showed even lower adjusted R²-values, indicating weaker spatial associations with neonatal mortality. The generally low adjusted R²-values suggest that although these factors contribute to spatial variations in neonatal deaths, additional unexamined variables or non-spatial factors may also play significant roles.

**Figure 5.**
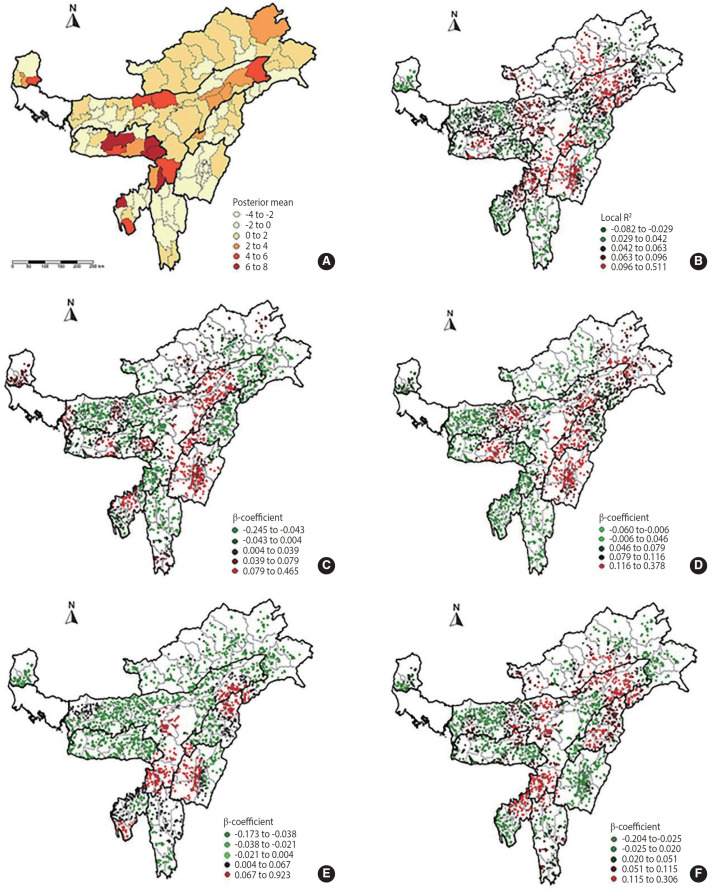
Cluster maps of the β–coefficient for factors influencing neonatal mortality across the north-eastern states of India. (A) Posterior means of the district-level random effect. (B) Local R2 for neonatal mortality. (C) Proportion of mothers with male infant. (D) Proportion of mothers with primary education. (E) Proportion of mothers with age above 30 years. (F) Proportion of mothers with less than 5 antenatal care visits.

Figure 5A displays a map of posterior means (point estimates) of district-level random effects. The map reveals a pattern of gradual change in point estimates between neighbouring districts, suggesting residual spatial variation unexplained by the current model. Districts with positive posterior means indicate higher probabilities of neonatal deaths, while districts with negative estimates have lower probabilities. Figure 5B illustrates local R²-values for neonatal mortality across all districts of the north-eastern states. Higher local R²-values were observed in the valley region of Manipur, upper and lower regions in Assam, and central regions of Arunachal Pradesh and Meghalaya. The local R²-values indicate that the model explains between 0.096 and 0.511 (approximately 9.6 to 51.1%) of the variation in neonatal mortality across Assam, Manipur, Meghalaya, Nagaland, and Arunachal Pradesh. Posterior means were computed using the Bayesian multilevel logistic regression model with integrated nested Laplace approximation, controlling for all independent variables (child, maternal, household, and community characteristics).

The GWR findings further demonstrated spatial clustering of neonatal mortality concerning selected predictor variables. The significance and direction of these correlations varied locally. GWR results pinpoint districts with the strongest regression coefficients, indicated by the darkest red colour on the map. A higher regression coefficient indicates a stronger local relationship between predictor variables and neonatal mortality. Figure 5C-F shows the regression coefficients of predictor variables significantly associated with neonatal mortality, including maternal age of 30 years or older, low levels of primary education, male infants, and less than 5 ANC visits. The GWR analysis specifically demonstrated that advanced maternal age was associated with higher neonatal mortality in most districts (Figure 5E), with the highest regression coefficients observed in the valley region of Manipur and southern regions of Assam.

## DISCUSSION

The present study found that neonatal mortality was more common in rural areas and among male infants. Mothers with advanced maternal age and those with only primary education were also significantly associated with neonatal mortality. Additionally, the analysis revealed higher neonatal mortality among households in the lowest wealth quintile and among mothers who had less than 5 ANC visits in the north-eastern states. These findings are consistent with spatial studies in other context, such as São Paulo, Brazil, where mother’s education and access to healthcare services were also associated with neonatal mortality [20]. A similar association between low household wealth, lower maternal education, and increased neonatal mortality was reported by Sidi-Yakhlef et al. [21]’s study conducted in Algeria. Furthermore, Singh et al. [22], in their research on rural India, showed that households with adequate sanitation facilities had lower NMRs. Consistent with our findings, Singh et al. [22] also noted that neonatal mortality was higher among male infants compared to females. This study specifically employed spatial analysis techniques to examine neonatal mortality and predictor variables within and across the north-eastern states of India. Spatial hotspot clustering was prominently observed in the eastern and southern regions of Assam, western and southern districts of Tripura, central regions of Meghalaya, and the southern region of Manipur. Additionally, the GWR analysis identified maternal age over 30 years, low maternal education levels, less than 5 ANC visits, and male infants as significant predictors of neonatal mortality, with regression coefficients varying considerably across districts. The study also revealed a positive association between advanced maternal age and neonatal mortality in most districts, particularly with high regression coefficients observed in the valley region of Manipur and the southern region of Assam [22].

Unlike previous studies, which primarily focused on national or rural-urban differences, this research expands on prior findings by integrating spatial and Bayesian multilevel modelling techniques to reveal region-specific risks and disparities. Our analysis provides detailed district-level insights into neonatal mortality, offering valuable information for policymakers.

### Strengths and limitations

This study provides a comprehensive analysis of neonatal mortality in the north-eastern states of India using advanced spatial modelling techniques. The use of Bayesian logistic regression enables a robust exploration of associations between neonatal mortality and various predictors, effectively accounting for potential multicollinearity. Identifying spatial heterogeneity and hotspot clusters provides valuable insights into regional disparities, informing targeted public health interventions. Additionally, the use of data visualisation facilitates clearer communication of the results to both policymakers and lay audiences, making it easier to identify regions requiring immediate and focused attention.

However, this research also has several limitations. Since NFHS data are self-reported, they may be subject to biases or inaccuracies due to potential concealment or recall errors by participants. The study’s accuracy is further constrained by the availability and precision of district-level data, which might impact the accuracy of spatial models. Although Bayesian methods offer considerable flexibility, they require careful specification of prior distributions, and inappropriate priors may introduce bias. Moreover, the cross-sectional nature of the data limits the ability to draw causal inferences from the observed associations.

## Figures and Tables

**Figure f6-epih-47-e2025021:**
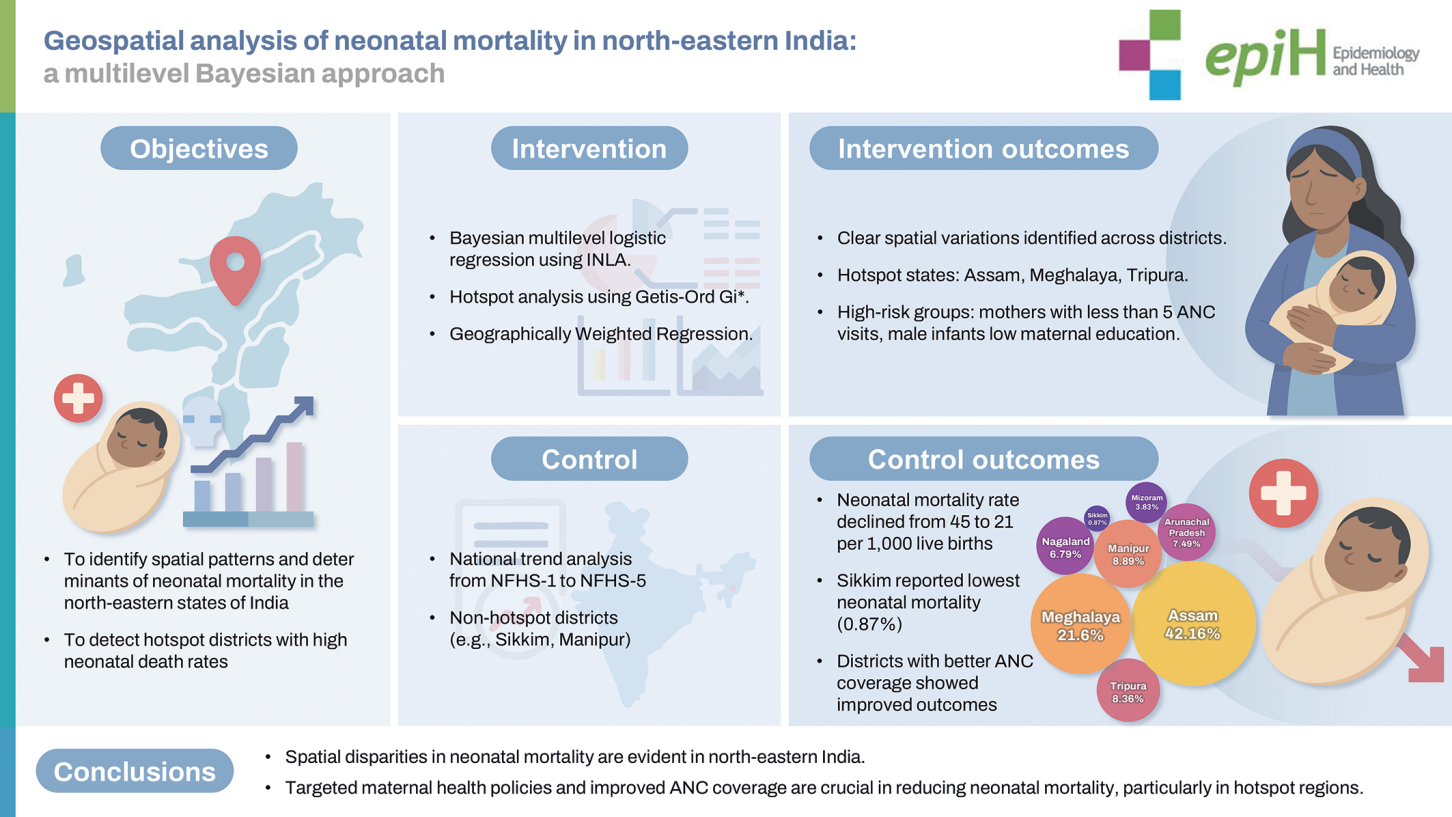


**Table 1.** Descriptive statistics and Bayesian multilevel logistic regression results for neonatal mortality in the north-eastern states

Variables	Sample		Neonatal mortality	
	% (total cases)	% (total cases)	p-value (χ^2^)	aOR (95% CI)^1^
Sex of child			<0.001	
Female	49.12 (16,810)	46.56 (223)		1.00 (reference)
Male	50.88 (17,412)	58.11 (351)		1.98 (1.63, 2.38)
Size of child (cm)			0.001	
Below	9.31 (3,185)	66.46 (109)		1.00 (reference)
Average	73.11 (25,020)	50.42 (363)		0.48 (0.35, 0.63)
Above	17.58 (6,017)	51.26 (102)		0.51 (0.36, 0.69)
Child’s birth order			0.080	
First	36.90 (12,629)	50.12 (201)		1.00 (reference)
Second	29.40 (10,061)	50.71 (143)		0.85 (0.66, 1.10)
Third	33.70 (11,532)	57.50 (230)		0.54 (0.41, 0.70)
Maternal age (yr)			<0.001	
15-19	3.27 (1,118)	28.00 (21)		1.00 (reference)
20-24	22.96 (7,856)	46.50 (146)		2.99 (2.43, 3.67)
25-29	33.55 (11,482)	51.24 (165)		5.17 (4.09, 6.37)
30-49	40.23 (13,766)	65.05 (242)		12.01 (9.46, 14.88)
Mother’s education level			<0.001	
Primary	33.50 (11,466)	60.73 (249)		1.00 (reference)
Secondary	58.87 (20,146)	50.74 (308)		0.95 (0.76, 1.16)
Higher	7.63 (2,610)	25.76 (17)		0.26 (0.17, 0.36)
Mother’s BMI (kg/m^2^)			<0.001	
Thin	12.09 (4,139)	66.29 (118)		1.00 (reference)
Normal	71.82 (24,577)	49.87 (386)		0.56 (0.42, 0.72)
Obese	16.09 (5,506)	53.44 (70)		0.69 (0.52, 0.92)
ANC visit			<0.001	
<5	74.08 (25,353)	60.10 (497)		1.00 (reference)
≥5	25.93 (8,869)	30.08 (77)		0.27 (0.20, 0.35)
Drinking water			0.160	
Safe	82.10 (28,095)	51.92 (447)		1.00 (reference)
Unsafe	17.90 (6,127)	57.21 (127)		1.13 (0.86, 1.51)
Wealth index			<0.001	
Low	33.34 (11,408)	60.39 (218)		1.00 (reference)
Middle	33.33 (11,407)	55.68 (201)		0.98 (0.79, 1.27)
High	33.33 (11,407)	42.94 (155)		0.60 (0.50, 0.73)
Place of residence			0.030	
Urban	14.84 (5,077)	43.23 (55)		1.00 (reference)
Rural	85.16 (29,145)	54.23 (519)		1.09 (0.87, 1.36)

aOR, adjusted odds ratio; CI, credible interval; BMI, body mass index; ANC, antenatal care.

1 Control variables: cooking fuel, sanitation facility, religion, ethnicity.
